# Heavy Drinking and Non-Medical Use of Prescription Drugs among University Students: A 9-Year Follow-Up

**DOI:** 10.3390/ijerph16162939

**Published:** 2019-08-16

**Authors:** Alicia Busto Miramontes, Lucía Moure-Rodríguez, Ainara Díaz-Geada, Socorro Rodríguez-Holguín, Montserrat Corral, Fernando Cadaveira, Francisco Caamaño-Isorna

**Affiliations:** 1 Department of Public Health, Universidade de Santiago de Compostela, Santiago de Compostela 15782, Spain; 2 CIBER de Epidemiología y Salud Pública (CIBERESP), Madrid 28029, Spain; 3 Department of Clinical Psychology and Psychobiology, Universidade de Santiago de Compostela, Santiago de Compostela 15782, Spain

**Keywords:** substance abuse, pharmacoepidemiology, university students, cohort

## Abstract

**Purpose:** Investigations suggest non-medical use of prescription drugs (NMUPD) is associated with heavy drinking and polydrug use among university students. Our aim is to determine the prevalence of NMUPD among university students and to analyze its association with alcohol, tobacco, and cannabis use, and to study the role of the age of drinking onset. **Methods:** Cohort study among university Spanish students (*n* = 1382). Heavy drinking (HED) and risky consumption (RC) were measured with the Alcohol Use Disorders Identification Test. Questions related to tobacco and cannabis consumption were also formulated. NMUPD refers to sedative, anxiety, or pain medication intake within the last 15 days without medical prescription. All variables were measured at 18, 20, and 27 years. Multilevel logistic regression for repeated measures was used to obtain adjusted OR (odds ratios). We analyzed the results from a gender perspective. **Results:** Prevalence of NMUPD were higher in students who already partook in NMUPD at the beginning of the study. NMUPD in women at 27 is 3 times higher than at 18, while in men it is twice. Among females, RC (OR = 1.43) and cannabis consumption (OR = 1.33) are risk factors for NMUPD, while later onset of alcohol use (OR = 0.66) constitutes a protective factor. No significant differences were found for males. **Conclusions:** NMUPD is prevalent among university students. RC and early onset of alcohol use were associated with higher prevalence of NMUPD in females. The prevalence of NMUPD increased with age in both sexes. Strategies for reducing risky drinking and delaying onset of drinking should be provided for university students. Pharmacists and parents should be alerted to the risk of NMUPD.

## 1. Introduction

Substance abuse in young people is a serious public health concern, especially in regard to cannabis and highly prevalent forms of alcohol risky consumption such as heavy episodic drinking [1,2]. Abuse of these substances has been associated with neurocognitive alterations in the still developing young brain [3,4] and also with other major social problems such as traffic accidents and unsafe sex [5,6].

In the last few years, pharmacoepidemiological studies involving university students have shown that the non-medical use of prescription drugs (NMUPD) is common in this population [7,8,9,10]. NMUPD has increased steadily among young people in the last decade (usually linked to self-treatment, experimentation, and readily accessibility), and the consequent incidence of unintentional overdoses has reached epidemic levels [11,12,13]. In addition, NMUPD is a risk factor for suicide [14,15], negative sexual behavior [16,17], and poor social functioning [18].

Recent studies indicate that NMUPD is associated with heavy drinking, alcohol problems, and polydrug use (e.g., including cannabis) [19,20,21]. In other studies, students who engaged in heavy drinking were between three and four times more likely to report NMUPD [19,20]. Similarly, in Spain, several studies have shown that high consumption of both medically prescribed and non-prescription medicines is associated with consumption of alcohol, tobacco, and cannabis among young people [22,23,24].

The epidemiological data on the association between NMUPD and alcohol, tobacco, and cannabis use among college students in Europe remains insufficient to date. Indeed, the lack of longitudinal studies hampers analysis of the changes in this association in the important developmental period between adolescence and adulthood, in which a decline in heavy drinking is expected [25]. Another aspect given little attention is the role of the age of drinking onset in this relationship, which given the tendency for young people to start drinking alcohol at an early age- deserves further attention in the context of NMUPD [6]. Findings regarding sex-related differences and this association are inconsistent, which may be partly explained by differences in the populations investigated, with some studies reporting a greater risk in females and others in males [26]. This is of particular importance considering that females may be particularly vulnerable to the long-term consequences of alcohol abuse [27]. Furthermore, accessibility, a key explanatory factor for the use of medical drugs, varies significantly between countries [9]. Further epidemiological studies are thus necessary to enable context-dependent conclusions to be drawn and transcultural extrapolations to be made. Ultimately, addressing these gaps may have important implications for public health prevention strategies by improving their focus.

The aim of this study was to determine the prevalence of NMUPD among university students in Spain and to analyze the association between NMUPD and the most prevalent patterns of alcohol, tobacco, and cannabis consumption among young adults, as well as the explanatory role of the age of drinking onset. We investigated this relationship by following the longitudinal trajectories of consumption in both males and females during the transition to adulthood. We also examined the potential variations in the long-term patterns of consumption in relation to whether or not the participants already partook in NMUPD at the beginning of the study, separately for males and females.

## 2. Materials and Methods

### 2.1. Design, Population, and Sample 

We carried out a cohort study among university students (Compostela Cohort 2005, Spain), between November 2005 and February 2015. We used cluster sampling to select the participants. Thus, at least one of the first-year classes was randomly selected from each of the 33 university faculties/schools (a total of 53 classes). The number of classes selected in each university faculty or school was proportional to the number of students. All students present in the class on the day of the survey were invited to participate in the study (*n* = 1382). This study was approved by the Bioethics Committee of the University of Santiago de Compostela. Subjects were informed both verbally and in writing, as part of the questionnaire, that participation was voluntary, anonymous, and the possibility to opt-out was available at any time. 

### 2.2. Data Collection Procedures

Researchers visited each first-year classroom in November 2005 and invited all students present in the class to participate in the study. Participants were evaluated via a self-administered questionnaire in the same classroom (1st questionnaire). In November 2007, the same team of researchers visited the third-year classroom in order to follow-up with the students. Participants were re-evaluated via a self-administered questionnaire (2nd questionnaire). The questionnaires were linked using birth date, sex, school, and class. Students who provided a phone number in the first or second questionnaire were further evaluated by phone after 9.0 years (March 2015). On all three occasions, alcohol use was measured with the Galician validated version of the Alcohol Use Disorders Identification Test (AUDIT) [28,29]. In addition to the AUDIT, we used another questionnaire that asked about age of onset of alcohol use, tobacco, and cannabis consumption, and use of medicines. The subjects were asked about their use of different medicines, with and without prescription, during the previous 15 days, using the Spanish National Health Survey [30]. More details about data collection are available in the following references [22,31].

### 2.3. Definition of Variables 

#### 2.3.1. Independent Variables

Heavy drinking: Dichotomous variable generated from the third AUDIT [28] question “How often do you have 6 or more alcoholic drinks per occasion?”, which was coded as follows: Never = 0, less than once a month = 0, once a month = 1, once a week = 1, daily or almost daily = 1. The sensitivity and specificity of this question with this cut-off value are respectively 0.72 and 0.73, and the area under the curve is 0.767 (95% CI: 0.718–0.816) [32].

Risky consumption: This (dichotomous) variable was generated from the AUDIT [28] score. A different cut-off value was established according to gender: ≥5 for women; and ≥6 for men. These cut-offs are recommended in the Galician validated version of the AUDIT [29]. 

Age of onset of alcohol consumption: Four categories were defined (after 16 years old, at 16, at 15, before the age of 15).

Cannabis consumption: Measured with the question “Do you consume cannabis when you go out? Never; Sometimes; Most of the times; Always” at the beginning of the study and at 2 years of follow-up. The categories were recategorized into No (“never”) and Yes (“sometimes”, “most of the time”, and “always”). At 9 years of follow up, cannabis consumption was measured using the European Addiction Severity Index (EuropASI) [33].

Tobacco consumption: Measured as a dichotomous variable: No/Yes at the beginning of the study and at 2 years of follow-up. At 9 years of follow up, we also used the European Addiction Severity Index (EuropASI) [33].

#### 2.3.2. Dependent Variable

Non-medical use of prescription drugs (NMUPD): Categorized as a dichotomous variable (“YES” refers to have taken sleeping medication/sedative or anxiety medication/stimulant medication/or pain medication in the past 15 days without medical prescription; and “NO” refers to the other cases. NMUPD criteria were adapted from those used by Boyd and colleagues [34].

### 2.4. Statistical Analysis

We used repeated measures multilevel logistic regression to obtain the adjusted odds ratios (OR) for independent variables in the use of medicines models. The 95% confidence intervals (95% CI) were calculated. These models are more flexible than traditional models and therefore allow us to work with correlated data, as we potentially have three measures of the same subject. Maximal models were generated, including all theoretical independent variables. Final models were generated from these maximal models. The final models included all significant variables or non-significant variables when their exclusion changed the OR of other variables by more than 10%. Data were analyzed using generalized linear mixed models in SPSS v.20 statistical software.

## 3. Results

The description of the samples at the beginning of the study for women and men are summarized in Table 1. There were no significant differences between samples for any variables.

The prevalence of risky consumption, heavy drinking, tobacco consumption, and cannabis consumption at baseline and after 2 years and 9 years are shown in Table 2. There was a significant reduction in the consumption of all substances and the specific patterns of consumption in the intervening period.

The proportion of NMUPD in relation to age of onset of alcohol use, risky consumption, heavy drinking, cannabis, and tobacco use at the ages of 18, 20, and 27 years is shown in Table 3. In general, the prevalence of NMUPD was greater among drug users (cannabis, tobacco, risky consumption, and heavy drinkers) than among non-users (tobacco use at age 20 for males whereas for females at age 18 risky consumption, heavy drinking, and cannabis; at age 20 risky consumption and cannabis, and at 27 risky consumption and tobacco). The prevalence of NMUPD was highest in females with risky alcohol consumption (Table 3) and tended to increase over time. In addition, the prevalence of NMUPD was higher in females who started to consume alcohol before the age of 15 than in those who began consuming alcohol after this age.

**Figure 1:**
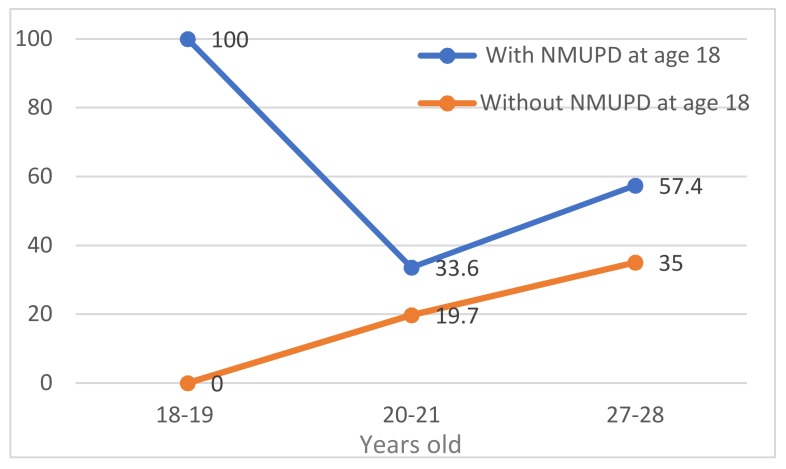
Trends in non-medical use of prescription drugs (NMUPD) (%) among women who already partook and who did not partake in this type of drug use at the beginning of the study.

**Figure 2:**
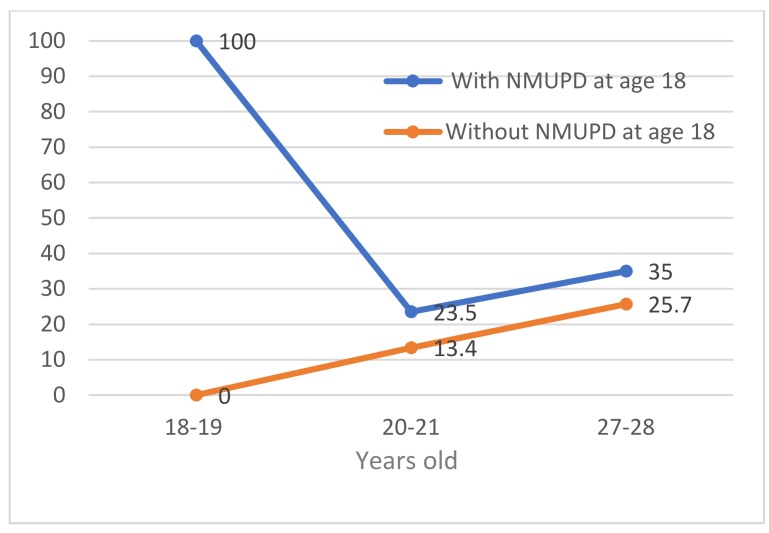
Trends in non-medical use of prescription drugs (NMUPD) (%) among men who already partook and who did not partake in this type of drug use at the beginning of the study.

The trends in the prevalence of NMUPD during the study period for students who had consumed and students had not consumed NMUPD at the beginning of the study are shown in Figure 1 (females) and Figure 2 (males). The prevalence rates were higher throughout the study in students who already partook in NMUPD at the beginning of study in both males and females. The prevalence of NMUPD was higher in both groups (already users or not) in both sexes in early adulthood (27–28 years old). At age 27 the difference between those who started university already partaking in NMUPD and those who did not partake in NMUPD was 22.4 percent points for females (see Figure 1) and 9.3 percent points for males (see Figure 2). Prevalence among those who practice NMUPD and those who do not were statistically significant among women at all ages (Figure 1) but were not among men (Figure 2).

Among females, the multivariate logistic regression models showed that risky alcohol consumption (OR = 1.43) and cannabis consumption (OR = 1.33) are risk factors for NMUPD. Later onset of alcohol use (at 16 OR = 0.63; after 16 OR = 0.66) constitutes a protective factor. Finally, the bivariate analysis revealed that a high frequency of heavy drinking is also associated with NMUPD (OR = 1.38). (Table 4).

Among males, there was no association between substance use or the age of drinking onset and NMUPD. The statistical models were rerun without including the stimulants medication for both sexes, and the findings did not differ. The final model also showed that the risk of consuming NMUPD increases with age in both sexes. The risk of incidence of NMUPD in women at age 27 is three times the risk at age 18 years, whereas in men the risk is twice as high for the same ages (Table 4).

## 4. Discussion

The aim of this study was to determine the longitudinal prevalence of NMUPD during the transition to adulthood in university students—taking gender into account—and how NMUPD is associated with patterns of tobacco, cannabis, and alcohol use (heavy drinking and risky consumption) and the effect of early drinking onset. The findings indicate that cannabis use and, in particular, risky alcohol consumption are risk factors for NMUPD among female students. Early onset of alcohol use was also a risk factor for NMUPD in females. However, no such association was found in males. Additionally, NMUPD was significantly higher at the age of 27, in both males and females.

Overall, the data revealed a high prevalence of NMUPD among the university students. Evidence from several studies has shown that non-medically prescribed drugs are readily accessible to young people, and the main sources seem to be family and friends [12,35,36,37]. The following reasons are often given to explain this type of drug abuse: For self-realization and recreational purposes, for relieving pain and anxiety, for combatting depression and for alleviating sleep-related problems [8,17,38]. Another context-dependent factor is that pharmacists do not seem to demand medical prescriptions from younger and/or more educated customers, such as university students [39].

Regarding sex-related effects, we observed striking differences between males and females. In males there was no association between substance use and NMUPD. On the contrary, in women, NMUPD is associated with risky consumption of alcohol (even heavy drinking in bivariate analysis). Risky drinking seems to be transculturally associated with NMUPD in young people [19,21,40,41,42,43]. Although some studies have reported a higher prevalence of NMUPD in males, many studies have indicated that females are at a greatest risk [9,26], (for a systematic review, see Young et al., 2012 [44]). Such differences may vary depending on the drugs investigated, the characteristics of the sample and the dose/frequency of drug use [26], although further studies are needed for confirmation. Excessive alcohol consumption may lead to dysregulation of the stress response, particularly in females [27], which hypothetically may contribute to a maladaptive coping style including the self-prescribed use of non-medically prescribed drugs to deal with emotional distress (as self-medication seems to be the most common motive in females [26] during adolescence). In addition to the cognitive alterations (e.g., memory deficits) that result from risky drinking during young adulthood, the possible interactions between these substances—together with the cognitive impairments associated with NMUPD [45]—may lead both to greater reinforcement of their addictive potential and to serious medical consequences [46,47]. 

Although higher prevalence of NMUPD among female cannabis users was found at the ages of 18 and 20, this did not translate onto an effect at the bivariable of multivariable analysis. This lack of association does not go in line with other authors’ results [19,20,21]. On the other hand, the lack of significant differences among NMUPD prevalence of female students who consume vs. do not consume cannabis at the age of 27 may be explained by the lower prevalence of cannabis consumption at this age—an association which may be difficult to find if it existed. 

Another variable which presented higher NMUPD prevalence among users, but did not show influence at the analyses, was tobacco consumption. Unlike other studies in our context found in scientific literature [22,23,24], tobacco use was not associated with NMUDP among college students, regardless of their gender. 

The lack of association of the consumption of these two substances with the NMUPD among university students should be studied in more depth. Future studies in our context should confirm or not these results, as well as look for possible explanations to them, since understanding not only what influences, but what does not influence NMUPD, allows the problem to be approached from a more effective perspective.

Our findings show how earlier age onset of alcohol consumption seems to increase the risk of practicing NMUPD. It is possible that some other factor is influencing this association, such as a predisposition to substance use, stress, or a certain personality, or even some combination of all of them. Having witnessed significant violence, suffering post-traumatic stress disorders, or having previous delinquent behaviors, having problems with family and friends, or having recent social environmental stressors have been related to NMUPD in university students by other authors, but further studies are necessary to support these hypotheses [48,49]. Nevertheless, delaying the age of drinking onset as a protective factor, has been previously referred in scientific literature [41,50]. Multiple hypotheses have been proposed to explain this association (e.g., the “gateway” hypothesis, the “common-model factor”) [51,52]. However, testing of these hypotheses is hampered by the lack of prospective studies beginning before drinking onset. This result adds support to a large body of literature highlighting the risks associated with early age onset of drinking, such as future psychopathological symptoms and alcohol dependence [53,54,55]. 

With respect to age-related changes, the prevalence of NMUPD in early adulthood (27 years) was higher than at younger ages (18 years) in both sexes, as also indicated in the Spanish National Health survey [30]. In females, the risk of NMUPD was three times higher at age 27 years than at age 18 years, and in men the risk was two times higher for the same ages. It is possible that NMUPD may increase with age [26], although few studies have covered the period of emerging adulthood. The fact that NMUPD increased throughout the study while other substance use decreased may be related to common transitions in social roles. Risky alcohol use in young people usually peaks during university years and decreases thereafter, probably due to the abandonment of the “campus alcohol culture” (peer pressure, availability/opportunity, normalization, etc.) and the acquisition of new adult roles linked to greater responsibilities, work, and family [31,56]. Conversely, greater social acceptance of NMUPD in adulthood and misconceptions about the safety of the drugs involved may partly explain the longitudinal trends observed in this study [57,58].

In this epidemiological study, a great effort was made to follow a large cohort of university students over a nine-year period. Nonetheless, the study is not without limitations. First, the use of self-reported questionnaires may lead to misrepresentation (under or over estimation) of the problem [59]. However, this type of test has been shown to produce reliable results when used with young adults and adolescents [60], and any misrepresentative results would probably affect descriptive rather than analytical findings [61]. Secondly, the loss of subjects over the follow-up period is a problem inherent to longitudinal designs and may lead to selection bias. However, the absence of significant differences between the initial sample and the follow-up sample suggests the lack of such bias.

## 5. Conclusions

NMUPD is prevalent among university students. Risky alcohol consumption and early onset alcohol use were associated with a higher prevalence of NMUPD in females, whereas no such association was found in males. The prevalence of NMUPD increased with age in both sexes. Prevention efforts should aim to educate university students regarding the potential effects of these drugs and their interactions. Strategies for handling stress during the university period should also be provided, with greater emphasis on females. In addition, pharmacists and parents should be alerted to the risk of NMUPD. Finally, the present study highlights the protective effect of delaying the age of drinking onset, beyond the typical alcohol-related problems.

## Figures and Tables

**Table 1:** Description of the samples at the beginning of the study according to the number of assessments carried out.

	Percentages							
	Women (992)				Men (371)			
	1 Time	2 Times	3 Times		1 Time	2 Times	3 Times	
	*n* = 323	*n* = 344	*n* = 325	*p*-Value	*n* = 165	*n* = 116	*n* = 90	*p*-Value
**Age of onset of alcohol use**								
After 16 years old	21.3	21.3	14.5		19.7	15.6	18.2	
At 16 years old	40.4	39.5	36.6		32.0	35.4	48.1	
At 15 years old	25.2	23.7	28.3		23.1	19.8	20.8	
Before age of 15 years	13.1	15.8	20.7	0.085	25.2	29.2	13.0	0.142
**Risky consumption**	51.7	48.3	54.8	0.241	61.2	55.2	55.6	0.522
**Heavy episodic drinking (%)**								
Never	61.0	62.5	60.0		33.9	41.4	45.6	
Less than once a month	20.7	19.5	22.5		31.5	19.8	21.1	
More frequently	18.3	18.0	17.5	0.919	34.5	38.8	33.3	0.123
**Cannabis consumption**	18.0	19.2	18.8	0.918	26.1	30.2	24.4	0.617

**Table 2:** Prevalence of alcohol, tobacco, and cannabis consumption at the beginning of the study, and at 2-year and 9-year follow-up.

	Percentage or Mean (95%CI)					
	Women			Men		
	Initial	2-Year	9-Year	Initial	2-Year	9-Year
**Risky consumption**	51.5	52.2	20.9	58.0	62.2	31.1
**Heavy drinking**						
Never	61.2	56.4	60.0	39.1	34.5	45.6
Less than once a month	20.9	26.9	22.5	25.3	26.7	21.1
More frequently	17.9	16.7	17.5	35.6	38.8	33.4
**Cannabis consumption**	18.6	16.1	4.0	27.0	19.9	8.9
**Tobacco consumption**	31.0	19.4	16.9	27.5	19.4	10.1

**Table 3:** Prevalence of non-medical use of prescription drugs at 18, 20, and 27 years old in relation to the substance use profiles of the subjects.

	Proportion of Subjects (%)					
	Females			Males		
	at 18 Year	at 20 Year	at 27 Year	at 18 Year	at 20 Year	at 27 Year
**Age of onset of use of alcohol**						
After 16 years old	12.2	19.2	40.0	15.5	10.3	35.7
At 16	17.0	19.0	36.6	16.9	14.1	16.2
At 15	23.5	24.7	43.6	8.7	17.1	31.2
Before the age of 15	26.8 *	27.6	45.6	18.7	15.8	40.0
**Alcohol risky consumption**						
No	15.8	18.4	37.0	14.7	16.9	29.0
Yes	21.7 *	26.1 *	50.5 *	15.3	14.0	25.0
**Heavy drinking**						
Never	16.3	21.2	36.2	15.9	16.9	35.1
Less than once a month	21.7	21.7	48.1	13.8	18.2	22.9
More frequently	24.2 *	27.7	43.8	15.2	11.2	22.2
**Cannabis use**						
No	17.1	20.9	39.7	14.4	15.2	30.5
Yes	26.5 *	30.6 *	38.5	17.0	14.6	
**Tobacco use**						
No	17.7	21.7	35.2	14.1	12.7	29.6
Yes	21.4	25.4	58.2 *	17.6	25.0 *	11.1
**Total**	18.9	22.4	39.7	15.1	15.0	27.8

* *p* < 0.05 between categories.

**Table 4:** Influence of age of onset of alcohol use and substance use in the non-medical use of prescription drugs. Logistic regression.

	Odds Ratio (95% Confidence Interval)		
	Females		Males
	Bivariate	Multivariate ^a^	Bivariate
**Age of onset of use of alcohol**			
Before the age of 15	1	1	1
At 15	0.85 (0.62–1.18)	0.89 (0.64–1.24)	0.66 (0.33–1.32)
At 16	0.59 (0.43–0.80)	0.63 (0.48–0.90)	0.81 (0.46–1.45)
After 16 years old	0.49 (0.33–0.72)	0.66 (0.42–0.94)	0.81 (0.40–1.62)
**Alcohol risky consumption**			
No	1	1	1
Yes	1.31 (1.06–1.62)	1.43 (1.10–1.86)	0.86 (0.57–1.30)
**Heavy drinking**			
Never	1		1
Less than once a month	1.27 (0.99–1.64)		0.89 (0.54–1.49)
More frequently	1.38 (1.04–1.84)		0.75 (0.46–1.22)
**Cannabis use**			
No	1	1	1
Yes	1.48 (1.13–1.93)	1.33 (0.99–1.81)	0.96 (0.59–1.58)
**Tobacco use**			
No	1		
Yes	1.23 (0.97–1.56)		1.36 (0.85–2.17)
**Age of the subjects**			
18 years-old	1	1	1
20 years-old	1.24 (0.98–1.56)	1.19 (0.91–1.55)	1.00 (0.62–1.61
27 years-old	2.83 (2.16–1.73)	3.45 (2.50–4.74)	2.16 (1.25–3.73)

^a^ Adjusted by all variables included in the column.
